# Influence of COVID-19 pandemic on colon cancer presentations

**DOI:** 10.3389/fonc.2026.1667288

**Published:** 2026-02-20

**Authors:** Arina Cipriana Pietreanu, Antonia Ioana Vasile, Cornel Cheregi, Simona Trifu, Bogdan Mihai Cristea, Bogdan Socea

**Affiliations:** 1 Doctoral School, Carol Davila University of Medicine and Pharmacy, Bucharest, Romania; 2 Department of Surgical Disciplines, Faculty of Medicine and Pharmacy, University of Oradea, Oradea, Romania; 3 Neurosciences Department, Carol Davila University of Medicine and Pharmacy, Bucharest, Romania; 4 Department of Anatomy, Carol Davila University of Medicine and Pharmacy, Bucharest, Romania; 5 Department of General Surgery, Carol Davila University of Medicine and Pharmacy, Bucharest, Romania

**Keywords:** cancer-related mortality, colon cancer emergencies, fear of COVID-19, metastatic cancer, the influence of pandemic on colon cancer patients

## Abstract

**Aim:**

We assumed that patients would present during the pandemic with more advanced, more disseminated, and more symptomatic forms of colon cancer.

**Materials and methods:**

We did a retrospective observational study that analyzed the database of the General Surgery Department of a tertiary care center in Romania, analyzing information about the patients from their clinical charts. The study was conducted on 204 patients diagnosed with colon cancer, subdivided into the pre-pandemic group (2019) and the pandemic group (2020). The ages varied between 27 and 95 years old, male and female. We measured: age, gender, year of admission, tumor localization, admission reasons, colon cancer emergencies, post-operative colon cancer complications, endoscopic tumor characteristics, histopathologic result, concomitant colonoscopy finding, presence and sites of metastases, and discharge status.

**Results:**

The number of presentations was lower in the pandemic year, but non-significant. During the pandemic, patients presented more with abdominal bloating, infiltrative tumors, multiple metastases, especially peritoneal metastases, indicating a more advanced local disease. As for colon cancer emergencies, inferior digestive hemorrhage occurred more frequently, consistent with more locally aggressive tumor behavior.

**Conclusions:**

During the pandemic, more patients presented with more oncologically advanced forms of colon cancer, but the cancer-related in-hospital mortality was not influenced. While the pandemic did not increase overall emergency presentations or short-term mortality, it was associated with delayed diagnosis and a higher burden of locally advanced and metastatic colon cancer at presentation.

## 1 Introduction

From the moment of the debut of the SARS-COV2 pandemic, all the sanitary systems had to make major changes in terms of medical organization of resources to be able to face the challenges imposed by the virus (1). The medical reorganization had to focus on keeping human resources and medical resources to treat patients who suffered from COVID-19 infection, and also had to focus on preventing intrahospital transmission of COVID-19 infection to other patients who suffered from other pathologies (2). In terms of surgical reorganizations, many elective surgeries were suspended (3), while acute emergencies were still operated as much as possible (4). Moreover, because of the global health crisis, many screening programs, including the one for colorectal cancer, have been suspended (5, 6). Non-urgent and non-cancer procedures were avoided to relocate nurses and anesthetists medical personnel to face the coronavirus patients (7).

The results from England explained that the pandemic had negative effects on screening programs for colorectal patients, on clinical presentation, and on treatment options for these patients (8). The authors demonstrated that the rate of early diagnosis of colorectal cancer has decreased, as many colonoscopies have been postponed or avoided, and surgeries have been postponed, to avoid the risk of contact with the coronavirus (8). The management used in Brazil implemented telemedicine to evaluate how many patients needed a face-to-face consultation and how many suggested benign diseases (9). In their territorial sanitary system, all benign diseases were postponed (9). The authors evaluated the safety of performing elective surgeries during the pandemic and planned 103 colorectal surgeries. Out of the 103 surgeries, 4 were avoided due to COVID-19 positivation of patients, 9 were for inflammatory bowel disease, and 90 were for colorectal cancer. Out of the operated patients, 5 have developed COVID-19 infection in the postoperative period, and 3 of them died in the intensive care unit. However, 95% of the patients who were operated on during the pandemic were discharged without any unfavorable consequences. The study concluded that elective colorectal surgeries can be performed safely during a pandemic; however, preoperative screening of patients is important to reduce the rate of intrahospital infection, given the high mortality rate of this virus infection (9). The importance of telemedicine during the pandemic was supported also by multiple previous studies (10). Challine et al. (11) evaluated differences in the management of colorectal patients before and during quarantine in France. Thus, in the pre-quarantine period, colonoscopies and colorectal resections were performed at a similar rate in the last 3 years. The authors identified a major change in the number of colonoscopies performed between March 2020 and May 2020. Comparing the number of colonoscopies performed in 2020 to those performed in 2019, there was a decrease of 26%. In the middle of the quarantine period (April 2020), the decrease reached 85%. A 34% decrease in screening colonoscopies was identified in patients with a family history of colorectal cancer. For colorectal resections, the authors found an 18% decrease from January to October 2020 compared to previous years. Surgical activity decreased by 33% in May 2020. These changes will lead to an increase in the number of undiagnosed colorectal cancers in the coming years (11).

At the beginning of the pandemic, postponing surgeries and cancelling medical appointments seemed like a viable medical option (12). O’Leary et al. (13) pointed out the changes in the treatment guidelines for colorectal cancers, emphasizing the appropriate recommendations according to the period of the pandemic. Therefore, surgeries on positive COVID-19 patients should be avoided. Urgent cases should be operated on if resources are available. According to established guidelines, elective surgery may be deferred and non-surgical treatment strategies initiated in patients with locally advanced rectal cancer or metastatic colorectal cancer (13). Urgent oncological cases can be delayed 6–12 weeks without threatening the oncological prognosis. O’Leary et al. (13) classified the period of the pandemic into three phases: phase 1 (semi-urgent situation where hospital resources are not exhausted and the trajectory of COVID-19 is not in a rapidly escalating phase), phase 2 (urgent situation where hospitals have many patients infected with COVID-19; the number of ventilators and blood banks have limited resources) and phase 3 (pandemic situation where all hospital resources are used or over capacity and are mainly directed towards the treatment of patients with COVID-19). Thus, in phase 1, urgent cases must be operated on (occlusive or subocclusive cancers, local or locoregional cancers, and early-stage rectal cancers that are not candidates for neoadjuvant therapy). In addition, rectal cancers with incomplete response to neoadjuvant therapy or selected cases with metastases should be operated on if they are at risk of worsening or chemotherapy has no longer benefits. In phase 2, all cases except emergencies should be postponed. In phase 3, surgeries must be restricted to acute emergencies; even emergencies are limited to those at high risk of mortality or morbidity without surgery, but who have a greater potential for recovery post-operatively. In comparison, an Italian guideline explained that the pandemic should not impair oncologic patients and expose these patients to higher morbidity, thus COVID-19-negative patients were to go under elective surgeries in COVID-19-free hospitals or in hospitals where positive and negative patients are clearly delimited (14).

Colon cancer signs and symptoms are generally nonspecific, becoming clinically apparent when the tumour is locally advanced (15). More than 15% of patients will present to the emergency room with acute colonic perforation or obstruction as the first sign of the disease (16). Colorectal emergencies may present with stricture, occlusion, perforation, or bleeding (17). Another previous study explained that patients who presented to the Emergency Department for colorectal cancer were the ones with more advanced tumors and with poorer stage distribution (18). Symptoms of colon occlusion include: abdominal pain, abdominal flatulence, constipation, and vomiting (17). Patients who present in more acute situations tend to have more frequent abdominal pain and vomiting, compared to patients who present themselves electively (19).

Colorectal emergencies are considered: acute obstruction, acute perforation, acute sepsis, and transfusion-requiring active bleeding (13). Urgent cases are considered: nearly obstructing tumors (subocclusion), perforation, fistulization, smouldering sepsis (13). Oncologically urgent cases include: stage I, II, and III of colon cancer, stage I rectal cancer, stage II/III rectal cancer after completed neoadjuvant treatment, and stage IV cancer that progresses with neoadjuvant therapy (13). All elective cases that may be postponed during the pandemic are: benign polyps, colitis with dysplasia, stoma reversal, and stage IV cancer with response to chemotherapy (13).

The aim of the study was to evaluate for differences between the clinical presentations of patients with colon cancer before the COVID-19 pandemic and during the COVID-19 pandemic in a tertiary care centre in Romania. To this end, the present study had 4 objectives: 1) characterization of the colon cancer patients depending on clinical presentation, colon cancer emergencies and post-operative colon cancer complications, associated comorbidities, and cancer subtypes; 2) evaluation of the difference in clinical presentation of patients before the pandemic and during the pandemic; 3) evaluation of the difference of colon cancer emergencies of patients before the pandemic and during the pandemic; 4) highlighting the impact of the pandemic on oncological staging and mortality for colon cancer patients.

## 2 Materials and methods

### 2.1 Study procedure and design

The study design was retrospective, observational, and cross-sectional.

The study retrospectively analyzed the database of the General Surgery Department from “St. Pantelimon” Hospital, in Bucharest, Romania. This hospital is a tertiary care centre in the capital city of Romania. However, it did not include an Oncology Department at the time of collecting data for this study. We analyzed information about the patients from their clinical charts and their discharge records.

Because the aim of our study was to compare the clinical presentations of patients before the pandemic and during the pandemic, we enrolled patients who were hospitalized in the General Surgery Department in the entire calendar year of 2019 and the entire calendar year of 2020. We chose these two periods because we wanted to evaluate the differences between the pre-pandemic period (January–December 2019) and the pandemic period (January–December 2020).

The year 2019 was selected as a reference interval, representing a baseline period during which the Romanian healthcare system functioned under normal conditions, without restrictions related to the SARS-CoV-2 outbreak.

The year 2020 was selected to represent the pandemic period, as it encompassed all major phases of the COVID-19 crisis in Romania. Chronologically speaking, the first reported case was in December 2019 in Wuhan, China (20). The cases started in China and afterwards were distributed among countries on every continent, but predominantly Japan, Thailand, Singapore, Hong Kong, Australia, and Taiwan. In Europe, the first cases were reported in Germany, France, Spain and Italy (21). Even though the first cases that were declared in Italy were two Chinese tourists who arrived in Milan in the 23^rd^ of January from Wuhan, in all of Italy, several studies confirmed that there were patients with severe respiratory symptoms that were undetected as having COVID-19 infection from December of 2019 (22). In addition, there were several cases that were confirmed to be COVID-19 infections that were hospitalized in December of 2019, before the routine COVID-19 testing began. For example, in a case report it was identified SARS-COV2 RNA in an oropharyngeal sample collected from a child (who was suspected to have measles) in early December 2019, approximately 3 months before the first officially identified case of COVID-19 in Italy (23). About the relation between Italy and Romania, there are over one million Romanian citizens living in Italy, making them the largest Romanian immigrant population outside Romania and Moldova. After the outbreak of COVID-19 in Italy, thousands of Romanian citizens who worked in Northern Italy returned to Romania (24). Some scientific studies also concluded that COVID-19 started to spread in Romania due to the return migration, through case importation from Italy (25). The first case of infection with COVID-19 was confirmed in Romania on 26^th^ of February 2020. However, in 16^th^ of March 2020 was declared lockdown in Romania. During the early stages of the COVID-19 pandemic, while it was not so distributed around the entire world, in early December 2019- February 2020, many people in Romania feared that Romanian citizens returning from Italy would bring the virus into the country. This fear was amplified by media coverage and public discourse, which portrayed returning migrants as potential carriers of COVID-19, given the severe outbreak in Italy and the large Romanian diaspora living there (26).

From a medical point of view, following the confirmation of the first COVID-19 cases in Romania in late February 2020, a national state of emergency was declared in March 2020, leading to profound reorganization of the healthcare system. During this period, multiple restrictive measures were implemented, including the suspension of elective surgical procedures, limitation of outpatient services, redirection of medical personnel toward COVID-19 care, and temporary closure or repurposing of hospital departments. These measures persisted, with variable intensity, throughout the year 2020, including during the subsequent state of alert.

We considered the entire year 2020 as a pandemic year, as it reflects a comprehensive assessment of the cumulative impact of the COVID-19 epidemic in Italy and the subsequent pandemic on oncological care in Romania. Although the COVID-19 pandemic was officially declared in Romania only in March 2020, public fear of accessing hospitals was already high in January and February 2020. This fear was largely driven by concerns that Romanian citizens returning from Italy, where the outbreak had already intensified, could introduce the virus into the country. As a result, many cancer patients, including those with colorectal cancer, postponed hospital visits despite the presence of symptoms, and elective oncological surgeries were delayed.

Another argument that led us to compare the full pre-pandemic year (2019) and the full pandemic year (2020) was about comparing the same timeframe. Restricting the analysis to a narrower timeframe (analysing 1 full year versus 10 months) would risk underestimating the full impact of the pandemic on colorectal cancer management. Also, comparing the same timeframe ensured methodological consistency, minimised seasonal bias, and enabled an accurate evaluation of changes in clinical presentation, disease severity, and patient outcomes attributable to the COVID-19 healthcare disruption. This approach is consistent with methodologies adopted in previously published international studies assessing the indirect effects of the COVID-19 pandemic on oncological care (20, 27).

### 2.2 Participants

The study was conducted on 204 patients diagnosed with colon cancer (subgrouped depending on tumor localization) from the General Surgery Department. The patients were enrolled from January 2019 to January 2021. The participants’ group was subdivided into: the pre-pandemic group (the patients who presented during 2019) and the pandemic group (the patients who presented during 2020). The ages of the patients varied between 27 and 95 years old. We analyzed both male and female patients.

The inclusion criteria were: age over 18, diagnosis of colon cancer and eligibility for surgery (curative, cytoreductive or palliative).

We enrolled patients who were admitted to the General Surgery Department and who did not previously know that they had colon cancer, but also patients who knew that they had colon cancer (and were operated on, but at the time of presentation had new symptoms, or were not operated on). Practically, we enrolled patients who were admitted with a suspected diagnosis of colorectal cancer based on their reason for hospitalization (including abdominal pain, altered bowel habits, abdominal bloating, nausea or vomiting, or rectal bleeding), and in whom colon cancer was subsequently confirmed after diagnostic investigations and postoperative examinations (colonoscopy, histopathology result). Only patients in whom the primary colonic tumor was considered resectable at the time of diagnosis were included, even in the presence of metastatic disease, with the intent of proceeding to surgical resection followed by adjuvant chemotherapy or radiotherapy as appropriate. Secondly, we included patients with a known diagnosis of colon cancer, either previously operated or not, who presented to the hospital for the same reasons for admission.

We excluded patients who presented for post-operative reevaluation after surgery, but did not have any residual symptoms. We excluded patients with rectal cancer, with benign lesions of the colon and patients who were non-operable. Therefore, we excluded patients with technically unresectable disease who were not candidates for upfront surgical treatment and required neoadjuvant chemotherapy or radiotherapy and were referred to palliative care in oncology hospitals.

Any aspect of the work covered in this manuscript has been conducted with the ethical approval of all relevant bodies (approved by the Ethics Committee of Carol Davila University of Medicine and Pharmacy, Bucharest, Romania, protocol code 12531/4.10.2021). Patients’ informed consent was obtained at the time of admission of every patient.

### 2.3 Variables

From patients’ medical records, we measured the following variables: age, gender, year of admission, tumor localization (cecum tumor, ascending colon tumor, liver flexure colon tumor, transverse colon tumor, splenic flexure colon tumor, descending colon tumor, and sigmoid tumor), admission reasons, medical comorbidities, colon cancer emergencies (occlusion, subocclusion, peritonitis, inferior digestive hemorrhage, or anemia), post-operative colon cancer complications (anastomotic fistula, anastomotic hemorrhage, abdominal adhesion syndrome, or splenic laceration), endoscopic tumor characteristics, histopathologic result, concomitant colonoscopy finding, presence of metastases and discharge status.

Admission reasons were identified from medical records and submitted as dichotomic variables as if the patient had or had not experienced the following symptoms: abdominal pain, altered bowel habits, abdominal bloating, nausea and/or vomiting, weight loss, deterioration of the general condition and rectal bleeding. Endoscopic tumor characteristics were objectified at the pre-operative colonoscopy and were submitted as: stenosing tumor, hemorrhagic tumor, infiltrative tumor, and ulcerated tumor. Histopathologic results divided patients into the following categories: poorly differentiated adenocarcinoma, moderately differentiated adenocarcinoma, well-differentiated adenocarcinoma, and mucinous colon carcinoma. Concomitant colonoscopy findings highlighted other colonic comorbidities such as hemorrhoidal disease, colon polyposis, and colon diverticulosis. Discharge status divided patients into those who were discharged and those who died during admission.

### 2.4 Statistical analysis

The statistical analysis was conducted in the SPSS program v.22.0. Statistical analysis was performed using appropriate tests according to data distribution. Categorical variables, including dichotomous variables, were compared using the Chi-square test or Fisher test. A p-value of <0.05 was considered statistically significant.

## 3 Results

### 3.1 Sample characteristics

The study analyzed 204 patients with colon cancer (92 female and 112 male). Depending on the year of admission and gender, the sample analyzed 126 patients pre-pandemic in 2019 (64 women and 62 men) and 78 patients during the pandemic in 2020 (28 females and 50 males). The ages of the sample varied between 27 and 95, with a mean age of 69.77 years (SD = 0.78).

Depending on tumor localization, the distribution was as follows: for the pre-pandemic year (2019): 18 patients with cecum tumor, 19 patients with ascending colon tumor, 12 patients with hepatic flexure tumor, 17 patients with transverse colon tumor, 13 patients with splenic flexure tumor, 12 patients with descending colon tumor and 35 patients with sigmoid tumor; while for the pandemic year (2020): 11 patients with cecum tumor, 14 patients with ascending colon tumor, 6 patients with hepatic flexure tumor, 8 patients with transverse colon tumor, 6 patients with splenic flexure tumor, 9 patients with descending colon tumor and 24 patients with sigmoid tumor. The Chi-square test demonstrated a non-significant difference between the two periods of time in terms of the number of presentations (p>0.05).

### 3.2 Impact of pandemic on clinical presentation of patients

The comorbidities that the patients had were: SARS-COV2 active acute infection, hypertension, cardiac comorbidities, dyslipidemia, type 2 diabetes, obesity, hepatic pathologies, hepatic steatosis, previous surgical abdominal interventions, history of colorectal cancer, gynaecological pathologies, breast cancer, smoking, alcoholism, presence of a stoma, gastric ulcer, thyroid pathology, neurological disorder, and rheumatological disease. The distribution of comorbidities between the two groups can be seen in **Table 1**.

**Table 1:** Distribution of comorbidities based on the year of admission.

Comorbidity	2019	2020	p-value
COVID-19 infection	0	4	0.020 (Fisher test)
Hypertension	65	36	0.451 (Chi-square)
Dyslipidemia	3	7	0.046 (Fisher)
Previous abdominal surgical interventions	51	30	0.775 (Chi-square)
Cardiac pathology	45	26	0.729 (Chi-square)
Gynaecological pathology	2	2	0.638 (Fisher)
Breast cancer	1	0	1 (Fisher)
Diabetus mellitus	26	13	0.484 (Chi-square)
Obesity	13	9	0.785 (Chi-square)
Alcoholism	4	2	1 (Fisher)
Presence of stoma	8	2	0.323 (Fisher)
Gastric ulcer	3	1	1 (Fisher)
Thyroid pathology	6	1	0.255 (Fisher)
Neurological disorder	10	6	0.950 (Chi-square)
Rheumatological disease	1	0	1 (Fisher)
Hepatic insufficiency	18	15	0.351 (Chi-square)
Hepatic steatosis	6	2	0.713 (Fisher)

When analyzing admission reasons, the distribution was as follows: for the pre-pandemic year (2019): abdominal pain (62 patients), altered bowel habits (56 patients), abdominal bloating (7 patients), nausea and/or vomiting (29 patients), weight loss (12 patients), deterioration of general condition (29 patients) and rectal bleeding (24 patients); while for the pandemic year (2020): abdominal pain (46 patients), altered bowel habits (28 patients), abdominal bloating (13 patients), nausea and/or vomiting (12 patients), weight loss (10 patients), deterioration of general condition (18 patients) and rectal bleeding (9 patients). To evaluate the impact of the pandemic on the clinical presentation of colon cancer patients, we ran a Chi-square test, and the results can be seen in **Table 2**. We demonstrated that the only symptom that alarmed the patient highly enough that would lead them to the hospital, even during the pandemic, was abdominal bloating.

**Table 2:** Chi-square test for clinical presentation of patients.

Clinical symptom	2019	2020	df	p-value	Chi-Square	Effect size
Abdominal pain	62/126	46/78	1	0.174	–	–
Altered bowel habits	56/126	28/78	1	0.228	–	–
Abdominal bloating	7/126	12/78	1	**0.010**	6.726	0.182
Nausea and/or vomiting	29/126	12/78	1	0.186	–	–
Weight loss	12/126	10/78	1	0.461	–	–
Deterioration of general condition	29/126	18/78	1	0.992	–	–
Rectal bleeding	24/126	9/78	1	0.157	–	–

Bold values mean statistically significant..

When analyzing colon cancer emergencies and post-operative colon cancer complications, the distribution based on the year of admission can be seen in **Table 3**. Comparisons between groups were performed using the chi-square or Fisher’s exact test, as appropriate. The analysis demonstrated that inferior digestive hemorrhage was the only variable that differed significantly between the two groups (p=0.047).

**Table 3:** Distribution of colonic cancer emergencies and post-operative colon cancer complications based on the year of admission.

Colon cancer emergencies and post-operative colon cancer complications	2019	2020	p- value
Occlusion syndrome	17 (13.49%)	17 (8.97%)	0.336 (Chi-square)
Subocclusion syndrome	23 (18.25%)	15 (19.23%)	0.863 (Chi-square)
Peritonitis	16 (12.69%)	13 (16.66%)	0.458 (Chi-square)
Inferior digestive hemorrhage	31 (24.6%)	11 (14.10%)	**0.047** (Chi-square)
Anemia	49 (38.88%)	36 (46.15%)	0.287 (Chi-square)
Abdominal adhesion syndrome	51 (40.47%)	33 (42.3%)	0.806 (Chi-square)
Ileus	8 (6.34%)	9 (11.53%)	0.167 (Fisher)
Anastomotic fistula	3 (2.38%)	1 (1.28%)	1 (Fisher)
Anastomotic hemorrhage	0 (0%)	1 (1.28%)	0.381 (Fisher)
Splenic laceration	0 (0%)	1 (1.28%)	0.381 (Fisher)

Bold values mean statistically significant..

### 3.3 Impact of pandemic on paraclinical presentation of patients

When analyzing endoscopic tumor characteristics identified at the pre-operative colonoscopy, the distribution based on year of admission can be seen in **Table 4**. As shown in **Table 4**, infiltrative tumors were more frequent during the pandemic year, showing a statistical difference (p=0.038).

**Table 4:** Distribution of endoscopic tumor characteristics based on the year of admission.

Tumor characteristics	2019	2020	p-value
Stenosing tumor	50 (39.68%)	29 (37.17%)	0.835 (Chi-square)
Hemorrhagic tumor	12 (9.30%)	8 (10.25%)	1 (Chi-square)
Infiltrative tumor	19 (15.07%)	21 (26.92%)	**0.038** (Chi-square)
Ulcerated tumor	21 (16.66%)	11 (14.10%)	0.771 (Chi-square)

Bold values mean statistically significant..

When analyzing concomitant colonoscopy findings (meaning other colonic pathologies identified at the pre-operative colonoscopy), the distribution based on the year of admission can be seen in **Table 5**. The statistical test demonstrated no significant differences between the two periods of time regarding associated colonic comorbidities identified on colonoscopy.

**Table 5:** Distribution of concomitant colonoscopy findings based on the year of admission.

Concomitant colonoscopy finding	2019	2020	p-value
Hemorrhoidal disease	24 (19.04%)	9 (11.53%)	0.160 (Chi-square)
Colon polyposis	6 (4.76%)	3 (3.84%)	1 (Fisher)
Colon diverticulosis	3 (2.38%)	2 (2.56%)	1 (Fisher)

When analyzing the histopathological result of the tumor, the distribution based on the year of admission can be seen in **Table 6**, which showed no statistically significant differences between the two periods of time.

**Table 6:** Distribution of histopathological results based on the year of admission.

Histopathological result	2019	2020	p-value
poorly differentiated adenocarcinoma	7 (5.55%)	7 (8.97%)	0.369 (Fisher)
moderately differentiated adenocarcinoma	41 (32.53%)	21 (26.92%)	0.405 (Chi-square)
well-differentiated adenocarcinoma	1 (0.79%)	1 (1.28%)	1 (Fisher)
mucinous colon carcinoma	7 (5.55%)	5 (6.41%)	0.781 (Fisher)

### 3.4 Impact of pandemic on colon cancer staging

We evaluated the impact of the pandemic on colon cancer staging, so we ran a Chi-square test for the variable of the presence of metastases at admission (independent of the localization of the metastases), and the results can be seen in **Table 7**. The difference is statistically significant (p=0.024), so we can conclude that the patients who presented during the pandemic had a more severe stage of colon cancer, with more cases presenting in stage IV cancer, compared to the patients who presented before the pandemic.

**Table 7:** Chi-square test for the presence of metastases in colon cancer patients.

Presence of metastases at admission	2019	2020	df	p-value	Chi-Square	Effect size
Yes	22/126	24/78	1	**0.024**	5.125	0.159

Bold values mean statistically significant..

When analyzing the metastasis sites at admission, the distribution based on the year of admission can be seen in **Table 8**. The test revealed that a statistically significant difference between the two periods of time was observed only for the presence of peritoneal metastases, which were more frequently identified during the pandemic year (p=0.021). No significant differences were found for other metastatic sites.

**Table 8:** Distribution of metastasis sites based on the year of admission.

Metastases	2019	2020	p-value
Hepatic	20 (15.87%)	16 (20.51%)	0.402 (Chi-square)
Pulmonary	4 (3.17%)	4 (5.12%)	0.704 (Fisher)
Peritoneal	2 (1.58%)	9 (7.14%)	**0.021** (Fisher)
Bone	1 (0.79%)	1 (1.28%)	1 (Fisher)
Pancreas	1 (0.79%)	0 (0%)	1 (Fisher)
Splenic	0 (0%)	2 (2.56%)	0.146 (Fisher)
Renal	1 (0.79%)	0 (0%)	1 (Fisher)
Suprarenals	0 (0%)	1 (1.28%)	0.381 (Fisher)
Epiploon	0 (0%)	2 (2.56%)	0.146 (Fisher)
Urinary bladder	0 (0%)	1 (1.28%)	0.381 (Fisher)

Bold values mean statistically significant..

### 3.5 Impact of pandemic on mortality of colon cancer patients

When analyzing discharge status, in 2019, out of 126 patients, 24 died (19.04%), while in 2020, out of 78 patients, 21 died (26.92%). Mortality was higher in 2020 compared to 2019 (26.9% versus 19.0%); however, this difference did not reach statistical significance (p > 0.05) (**Table 9**).

**Table 9:** Chi-square test for mortality of patients.

Discharge status	2019	2020	p-value
Discharged	102/126	57/78	0.22 (Fisher)
Died	24/126	21/78	

## 4 Discussions

The present study did a comparative analysis of the clinical and paraclinical presentation of colon cancer patients during the COVID-19 pandemic and before the pandemic. We highlighted the differences in terms of the number of patients that presented to the General Surgery Ward before and during the pandemic. We highlighted the differences in terms of clinical presentation of patients before and during the pandemic, focusing on admission reasons, colon-cancer emergencies and post-operative colon cancer complications. We highlighted differences between the paraclinical details of the patients (colonoscopy findings, histopathological results, number and sites of metastases). Lastly, we compared the mortality rate of colon cancer patients during the pandemic with that of the period before the pandemic.

The basis of our research was that the pandemic made some changes worldwide and in our country in terms of treating surgical cases, which led to multiple suspensions of elective surgeries (3). Acute urgent surgical cases and oncologic cases were still performed (4). Patients with malignancies and older adults were at a higher risk of contracting SARS-COV2 infection (28, 29). On the other hand, the failure of receiving regular cancer treatment increased the risk of cancer-related morbidity, complications, and mortality (30). Our research started from the hypothesis that colon cancer patients will present in more complicated stages of colon cancer during the pandemic compared to the period before the pandemic.

In terms of clinical presentation of patients with colon cancer before and during the pandemic, the first result of our study was that the number of presentations was lower in the pandemic year compared to the year before the pandemic, but with a non-significant difference. This result is concordant with the previous studies, which highlighted that patients had to focus on two significant fears about the hospital: the fear of contacting SARS-COV2 infection and the fear of being diagnosed with cancer (31). Understanding fear, anxiety, and panic about COVID-19 infection is essential for investigating mental health issues (32, 33). If we refer to the symptoms that determined the patients to arrive at the hospital, our study showed that in the pandemic, they presented especially the following symptoms: abdominal pain, abdominal bloating, weight loss, and deterioration of the general condition. However, the only symptom that differed between the two periods of time was abdominal bloating. In other words, during the pandemic, there were more patients who presented with abdominal bloating, a symptom that led the patient to the hospital, no matter the fear of contracting SARS-COV2 infection. Abdominal bloating is often associated with progressive luminal narrowing, impaired intestinal transit, or early obstructive phenomena, which tend to develop as the disease advances (34).

In terms of colon-cancer emergencies that led the patient to the Emergency Room during the pandemic, the patients presented more with: subocclusion, peritonitis, anemia, abdominal adhesion syndrome, and ileus. This result was also suggested by previous studies conducted on the European population, in the Netherlands, where the authors demonstrated that the COVID-19 pandemic led to more clinical presentations with ileus (5). This finding was in direct relation to our hypothesis, which was whether the colon cancer patients would present during the pandemic with more cancer-related surgical emergencies (such as occlusion, subocclusion, hemorrhage, anemia, ileus, or peritonitis). However, our study demonstrated that there were no significant differences in terms of presentations in acute surgical emergencies for colon patients during the pandemic, compared to the period before the pandemic, except for inferior digestive hemorrhage. In other words, this result is important because it highlights the fact that when a colon cancer patient gets to the medical specialist with a complicated form of colon cancer, he will present himself to the hospital no matter the medical environment (in our case independent of the pandemic worldwide situation). This result was similar to the previous studies performed in Spain which explained that the number of patients that needed emergency surgery remained the same (35). Although overall healthcare utilization decreased during the COVID-19 pandemic, previous studies have shown that patients with higher acuity conditions continued to present to emergency departments, even when lower-acuity visits were deferred. This suggests that true acute presentations, such as cancer-related emergencies, may have been less affected by pandemic-related healthcare avoidance behaviors, just like our study demonstrated (36). In addition, the higher rate of inferior digestive hemorrhage reflects more advanced and locally aggressive tumor characteristics at presentation. Tumor-related bleeding is often associated with mucosal ulceration, friability, and infiltrative growth, which can develop as the disease progresses (37). During the COVID-19 pandemic, delayed diagnostic evaluation and postponed medical consultation may have allowed tumors to evolve locally before presentation, increasing the likelihood of bleeding complications. In this context, inferior digestive hemorrhage may represent one of the clinical manifestations of delayed presentation rather than an increase in overall emergency presentations.

In terms of post-operative colon cancer complications, we did not find any significant differences between the wo periods of time. However, surgical site infection (SSI) remains one of the most common and clinically significant complications following colorectal surgery, largely due to the inherent bacterial load of the colon and the complexity of these procedures. Despite advances in perioperative care, colorectal procedures continue to be associated with higher SSI rates compared to other abdominal surgeries. Contributing factors include contamination of the operative field, prolonged operative time, emergency surgery, and patient-related risk factors such as advanced age, malnutrition, obesity, diabetes, and immunosuppression. The occurrence of SSI is associated with increased postoperative morbidity, prolonged hospital stay, delayed recovery, and higher healthcare costs. Moreover, SSIs can adversely affect oncological outcomes by delaying adjuvant therapy and increasing the risk of postoperative complications. Based on statistical internal reports of the hospital, the incidence rate in the surgery department is 1.62% in 2019 and decreased to 0,81% in 2020 due to systematic measures taken during the pandemic. Butyrylcholinesterase (BChE) plays a role as an inflammatory and metabolic biomarker in SSI. A previous study showed that patients with lower postoperative BChE levels had a significantly higher risk of developing SSI after colorectal surgery (38). Therefore, BChE may be a useful early indicator for identifying patients at increased risk of postoperative infectious complications.

In terms of paraclinical presentation of patients with colon cancer before and during the pandemic, we observed that at the macroscopic examination at the preoperative colonoscopy, during the pandemic year, the patients presented more with hemorrhagic tumors and infiltrative tumors. However, the only macroscopic characteristic of the tumor that made a difference between the two periods of time was the infiltrative type of tumor. During the pandemic, more patients presented with more infiltrative tumors which led them to the hospital. This result is consistent with the clinical finding about the abdominal bloating, reflecting a more advanced local disease at presentation. Infiltrative growth patterns are often associated with prolonged tumor progression, deeper mural invasion, and circumferential spread, which may develop when early symptoms are overlooked or when medical evaluation is postponed (39).

In terms of colon cancer staging, in accordance with our hypothesis, we investigated whether the pandemic period was associated with a higher proportion of patients presenting with more advanced or disseminated colorectal cancer. This hypothesis was confirmed, thus more patients presented with more oncologically advanced forms of colon cancer during the pandemic. When analyzing the metastases sites, during the pandemic, there were more patients who had multiple metastases (hepatic, pulmonary, peritoneal, in the bones, splenic, in the suprarenals, and in the urinary bladder). However, the only significant difference between the sites of metastases between the two years remained the peritoneal metastases. The higher proportion of patients presenting with metastatic disease during the pandemic period likely reflects delayed diagnosis and prolonged tumor progression prior to hospital admission. Among metastatic sites, the predominance of peritoneal involvement may be explained by the natural history of locally advanced colon cancer, in which progressive transmural invasion facilitates peritoneal dissemination. Peritoneal metastases are often associated with advanced local disease and infiltrative growth patterns, which may develop when diagnostic evaluation and treatment are postponed (40). During the COVID-19 pandemic, reduced access to diagnostic procedures, delayed colonoscopy, and patients’ reluctance to seek medical attention for non-acute symptoms may have allowed tumors to progress locally, increasing the likelihood of peritoneal spread before diagnosis.

Lastly, in terms of mortality, also in accordance with our hypothesis, we investigated whether the pandemic period was associated with a higher proportion of patients who died during admission. This hypothesis was infirmed, as the number was similar, with a non-significant difference. The mortality rate reported in the study (approximately 19% in 2019 and 26% in 2020) can be explained by a combination of advanced disease stage at presentation, delayed diagnosis, and increased disease severity, all of which were accentuated during the COVID-19 pandemic. Advanced-stage colorectal cancer is well known to be associated with increased perioperative mortality and reduced survival, independent of treatment quality (41). The COVID-19 pandemic (or any other pandemic) further exacerbated these risks by delaying patient presentation, limiting access to diagnostic procedures, and disrupting routine oncological pathways. Additionally, healthcare system reorganization, including the redistribution of medical resources and restricted availability of intensive care services, may have adversely affected perioperative management and postoperative recovery. Collectively, these factors provide a coherent explanation for the elevated mortality observed in this cohort and underscore the indirect but profound impact of the COVID-19 pandemic on outcomes of patients with colorectal cancer.

From another point of view, several studies published in Romanian medical journals have highlighted the significant psychological burden experienced by patients undergoing colorectal cancer surgery. Research from Romanian surgical and oncological centres has shown that anxiety, depressive symptoms, and emotional distress are common among patients diagnosed with colorectal malignancies, particularly in those undergoing major abdominal surgery or presenting with advanced disease stages (42). These psychological disturbances are often associated with impaired quality of life, delayed recovery, and reduced adherence to postoperative treatment. Romanian studies have also emphasized that mental health assessment is not routinely integrated into standard perioperative care, despite evidence that psychological distress may negatively influence postoperative outcomes and rehabilitation. This issue is further accentuated in patients undergoing emergency surgery or those requiring stoma formation, where emotional distress and adaptation difficulties are more pronounced (42). Additionally, limited access to structured psycho-oncological support within the national healthcare system has been identified as a contributing factor to underdiagnosis and undertreatment of mental health disorders in this patient population.

Colorectal cancer represents a major global health problem, with late diagnosis being a key factor that complicates treatment and increases mortality. Screening tests are critical because the disease is often asymptomatic in early stages, allowing early detection and treatment when outcomes are much better. A previous article shows that although healthcare workers in several Greek hospitals are expected to be well informed about colon cancer screening due to their role in patient care, significant gaps persist in both their knowledge of screening methods and their personal participation in such preventive practices (43). Improving awareness, education, and participation among healthcare staff could not only benefit their personal health but also strengthen their role as advocates for preventive screening in the general population. Promoting better understanding and adherence to recommended screening guidelines within the healthcare workforce may enhance overall prevention efforts and reduce the burden of colorectal cancer in the community.

The first limitation came from the observational and retrospective design of the study. The transverse study design implied a single time point of patient evaluation, which did not allow assessment of long-term outcomes. Therefore, conclusions regarding survival, recurrence, or oncological efficacy over time cannot be drawn. Recent studies with longer follow-up, including analyses of patients managed with curative intent during the COVID-19 lockdown and evaluated at three years, have shown that delayed diagnosis and treatment may influence medium- and long-term oncological outcomes (44). Future longitudinal studies with extended follow-up are needed to better define the long-term impact of the pandemic on colon cancer prognosis. Another limitation related to the study design was that it did not allow for a more detailed temporal analysis. A potential future research direction would be to investigate differences in the presentation patterns of patients with colorectal cancer across clearly defined time periods in relation to the pandemic. These periods could include a pre-pandemic phase, a phase characterized by a state of alert before the official declaration of the pandemic in Romania (or other countries), when the epidemic was already ongoing in other countries (which was the period of January and February 2020 in Romania), the pandemic period itself, and a post-pandemic period. Such an approach could provide a clearer understanding of how different phases of the pandemic influenced patient behavior and access to oncological care. Another limitation came from the retrospective study design, where we analyzed only clinical charts and discharge records of the patients. This way, there were some charts that did not have all the necessary data for the analysis (some patients did not have the histopathological result, some did not have a pre-operative colonoscopy, we did not have tumor dimensions for all patients, etc.). Another limitation in relation to missing data was from the patients who presented in very serious conditions that rapidly led to death, so we could not collect all relevant data (histopathological results, dissemination grade, etc.). In addition, for the severe cases that eventually led to death, we could not take the entire medical history and clinical symptoms. Another important aspect that we did not analyze was the imaging results, as we did not take into account CT or MRI findings. Another limitation came from the inclusion of both newly diagnosed patients and patients with recurrent colon cancer, which may have introduced heterogeneity. Future studies could focus on one of these patient groups to allow for a more homogeneous analysis. Another limitation came from not taking into account treatment options: we did not analyze differences depending on the consequences of surgery (total resection, partial resection, stoma, complications, etc.), and we also excluded patients who were not-operable and were redirected directly towards oncology. The main aim of our study was to evaluate differences in terms of clinical presentations and how the symptoms were correlated with the consequences. Another limitation of this study is that we did not perform a more detailed evaluation of patients’ discharge status, limiting the analysis to a binary classification of discharged or deceased. A potential future research direction would be to assess discharge status in a more refined manner, according to the type of surgical intervention and subsequent oncological management required. For example, patients could be categorized as: recovered (in cases of localized disease without lymph node involvement in which the primary tumor was completely resected); improved (when the primary tumor was resected but adjuvant or neoadjuvant therapy was required due to lymph node involvement or metastatic disease); worsened (in cases requiring transfer to the intensive care unit); or deceased. Such an approach could provide a more comprehensive understanding of postoperative outcomes and early disease trajectories.

## 5 Conclusions

Our study presented the impact that the COVID-19 pandemic had on the presentation of patients with colon cancer in a tertiary care center in Romania. The main hypothesis of our study assumed that the patients would present during the pandemic with more advanced, more aggressive, more disseminated, and more symptomatic forms of cancer. During the COVID-19 pandemic, fewer patients presented for colon cancer evaluation, having in mind the fear of contracting COVID-19 infection. Abdominal bloating was the only clinical symptom that differed significantly, while overall colon cancer–related emergencies remained unchanged, except for a higher frequency of inferior digestive hemorrhage. Tumors diagnosed during the pandemic more frequently exhibited infiltrative features, and patients presented with more advanced disease, characterized by a higher rate of metastatic involvement, particularly peritoneal metastases. Despite these changes in disease presentation and staging, in-hospital mortality was not significantly affected.

## Data Availability

The raw data supporting the conclusions of this article will be made available by the authors, without undue reservation.
